# Crystal structure of a supra­molecular lithium complex of *p*-*tert*-butyl­calix[4]arene

**DOI:** 10.1107/S2056989018001834

**Published:** 2018-04-17

**Authors:** Manabu Yamada, Muniyappan Rajiv Gandhi, Kazuhiko Akimoto, Fumio Hamada

**Affiliations:** a Research Center for Engineering Science, Graduate School of Engineering Science, Akita University, 1-1 Tegatagakuen-machi, Akita 010-8502, Japan; b Graduate School of International Resource Sciences, Akita University, 1-1 Tegatagakuen-machi, Akita 010-8502, Japan; c Nissan Chemical Industries, LTD, 6903-1 Ooaza-Onoda, Sanyo-Onoda, Yamaguchi 756-0093, Japan; d Emeritus Professor, Akita University, 1-1 Tegatagakuen-machi, Akita 010-8502, Japan

**Keywords:** crystal structure, calix[4]arene, supra­molecular lithium complex, inclusion compound, hydrogen bonding, C—H⋯π inter­actions

## Abstract

Crystals of a supra­molecular lithium complex with a calix[4]arene derivative, namely tetra­methano­llithium 5,11,17,23-tetra-*tert*-butyl-25,26,27-trihy­droxy-28-oxidocalix[4]arene methanol monosolvate, [Li(CH_3_OH)_4_](C_44_H_55_O_4_)·CH_3_OH or [Li(CH_3_OH)_4_]^+^·(calix[4]arene^−^)]·CH_3_OH (where calix[4]arene^−^ represents a mono-anion species because of deprotonation of one H atom of the calixarene hy­droxy groups), were obtained from *p*-*tert*-butyl­calix[4]arene reacted with LiH in tetra­hydro­furan, followed by recrystallization from methanol. The asymmetric unit comprises one mono-anionic calixarene mol­ecule, one Li^+^ cation coordinated to four methanol mol­ecules, and one methanol mol­ecule included in the calixarene cavity. The calixarene mol­ecule maintains a cone conformation by intra­molecular hydrogen bonding between one phenoxide (–O^−^) and three pendent calixarene hy­droxy groups (–OH). The coordinated methanol mol­ecules around the metal cation play a significant role in forming the supra­molecular assembly. The crystal structure of this assembly is stabilized by three sets of inter­molecular inter­actions: (i) hydrogen bonds involving the –OH and –O^−^ moieties of the calixarene mol­ecules, the –OH groups of the coordinated methanol mol­ecules, and the –OH group of the methanol mol­ecule included in the calixarene cavity; (ii) C—H⋯π inter­actions between the calixarene mol­ecules and/or the coordinated methanol mol­ecules; (iii) O—H⋯π inter­actions between the calixarene mol­ecule and the included methanol mol­ecule.

## Abstract 2

The crystal structure of a supra­molecular lithium complex of *p*-*tert*-butyl­calix[4]arene has been determined and analyzed. Different from the majority of calixarene–alkali metal complexes, which are formed by direct coordination of the metal cation to the calixarene hy­droxy groups, this complex is stabilized by an inter­play of weak inter­actions involving the methanol mol­ecules surrounding the metal, giving rise to a second-sphere coordination supra­molecular assembly.

## Chemical context   

Calixarenes are synthetic macrocyclic compounds that are composed of phenol rings, linked with methyl­ene groups at linking positions (Gutsche, 1998 ▸). They are versatile mol­ecules for the inclusion of organic and/or inorganic compounds into their flexible cavities and for the coordination of organic/metal ions in mol­ecular recognition phenomena and host–guest chemistry (Vicens & Böhmer, 1991 ▸). The coordination chemistry of alkali metal cations, involving conventional calixarenes (and their corresponding functionalized derivatives) as ligands, has been intensively investigated in the past years, as a possible method of selective extraction of this class of cations using calixarenes as extractant. At the same time, the X-ray analysis of alkali metal complexes with *p*-*tert*-butyl­calix[4]arene in the crystalline state has been reported (Bock *et al.*, 1995 ▸; Davidson *et al.*, 1997 ▸; Dürr *et al.*, 2006 ▸; Gueneau *et al.*, 2003 ▸; Guillemot *et al.*, 2002 ▸; Hamada *et al.*, 1993 ▸; Hanna *et al.*, 2002 ▸, 2003 ▸; Harrowfield *et al.*, 1991 ▸; Lee *et al.*, 2009 ▸). In the majority of cases, the alkali metal complexes of *p*-*tert*-butyl­calix[4]arene in the solid state show direct coordination of the metal ions to the oxygen atoms belonging to the calixarene hy­droxy groups at the lower rim, with the resulting crystal structures stabilized by weak inter­actions with the lattice solvent mol­ecules.
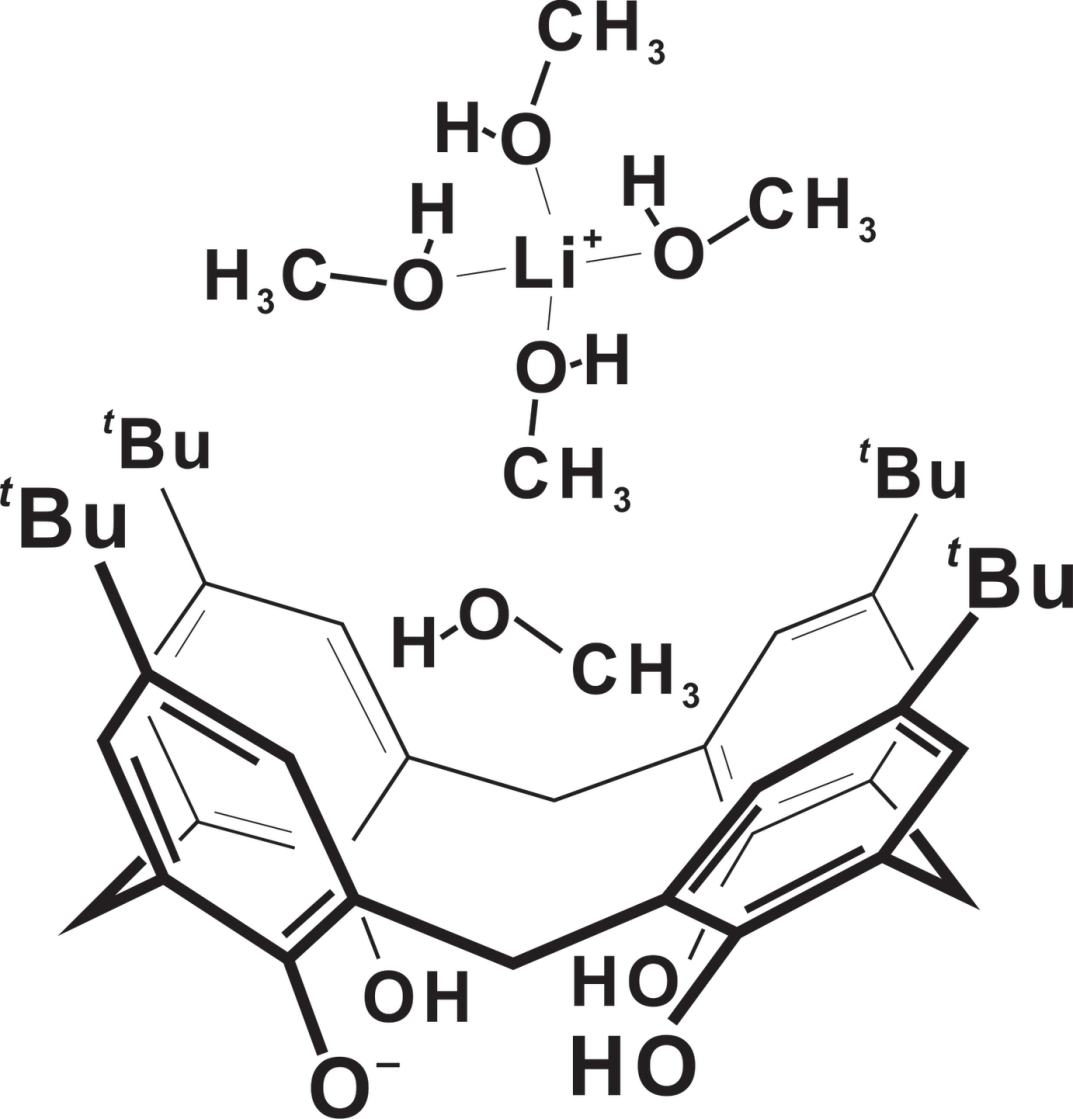


In the present paper, we report a different type of Li complex with *p*-*tert*-butyl­calix[4]arene, in which no direct coordination of the metal to the oxygen atoms of the calixarene hy­droxy groups takes place. The lithium cation is instead surrounded by four methanol solvent mol­ecules, which are in turn connected to the host mol­ecule *via* a series of hydrogen bonds, playing a significant role in the formation of the supra­molecular assembly.

## Structural commentary   

**Figure 1:**
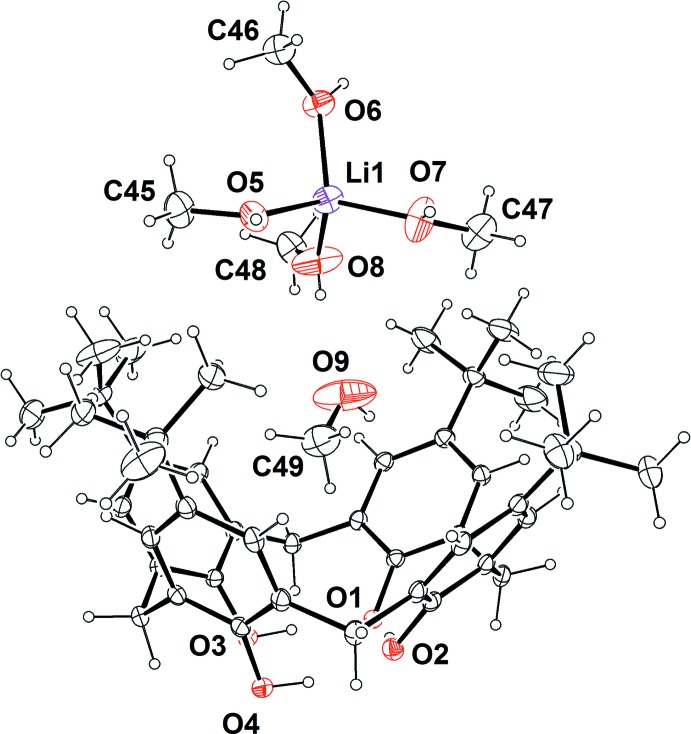
*ORTEP* diagram of the Li complex of *p*-*tert*-butyl­calix[4]arene with displacement ellipsoids at the 20% probability level.

Fig. 1 ▸ shows the mol­ecular structure of the complex [Li(CH_3_OH)_4_]^+^·(calix[4]arene^−^)]·CH_3_OH, consisting of one mono-deprotonated calix[4]arene unit in a cone conformation, one methanol mol­ecule included in the cavity, and one Li cation coordinated to four methanol mol­ecules. The positive charge of the methanol–lithium complex naturally dictates that the calixarene is in a mono-anionic form. The conformation of the macrocycle is stabilized by intra­molecular hydrogen bonding involving one deprotonated –O^−^ and three –OH groups at the lower rim, as shown in Table 1 ▸. The geometrical parameters of the cone conformer are given in Table 2 ▸, which reports the angle between the mean plane passing through the oxygen atoms O1, O2, O3 and O4, and the four mean planes passing through the aromatic walls (plane *A*: C1–C6/O1; plane *B*: C7–C12/O2; plane *C*: C13–C18/O4; plane *D*: C19–C24/O3). From these values, it is possible to notice that the two neighboring aromatic rings (C1–C6 and C7–C12) are slightly outward with respect to the other two adjacent aromatic moieties. Selected bond distances and angles for the tetra­kis­(methanol)–lithium complex are reported in Table 3 ▸.

**Figure 2:**
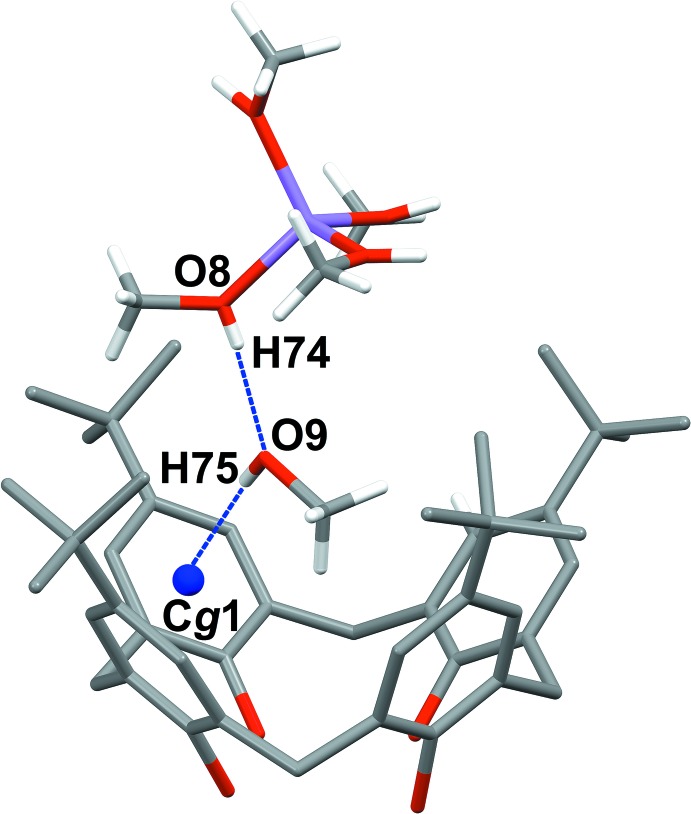
Hydrogen bonds (blue dotted lines) involving the *p*-*tert*-butyl­calix[4]arene anion, the methanol mol­ecule included in the cavity, and the [Li(CH_3_OH)_4_]^+^ complex belonging to the asymmetric unit. The centroid of aromatic the ring, *Cg*1, is represented as a blue sphere. The H atoms of the calixarene host have been omitted for clarity.

As shown in Fig. 2 ▸, one methanol mol­ecule is included in the cavity, displaying a short O—H⋯π inter­action involving the hy­droxy moiety and π-electrons of the calixarene aromatic ring C1–C6. The O9⋯*Cg*1 and the H75⋯*Cg*1 distances are 3.360 (6) and 2.538 (5) Å, respectively, while the angle O9—H79⋯*Cg*1 is of 166.34 (6)° (*Cg*1 is the centroid of the C1–C6 ring). On the other hand, there are no C—H⋯π inter­actions between the embedded methanol and the aromatic-π electrons of the calixarene, hence the included solvent is stabilized inside the calixarene cavity only by the O—H⋯π inter­action.

## Supra­molecular features   

The relevant feature of the title complex is that the lithium cation is not directly coordinated to the hy­droxy groups of the lower rim of the calix[4]arene host. On the contrary, the inter­action of the [Li(CH_3_OH)_4_]^+^ complex with the macrocycle in the asymmetric unit is mediated by the methanol mol­ecule embedded in the cavity, which acts as hydrogen-bond acceptor for a methanol mol­ecule (C48–O8) coordinated to the lithium cation (Fig. 2 ▸ and Table 1 ▸).

**Figure 3:**
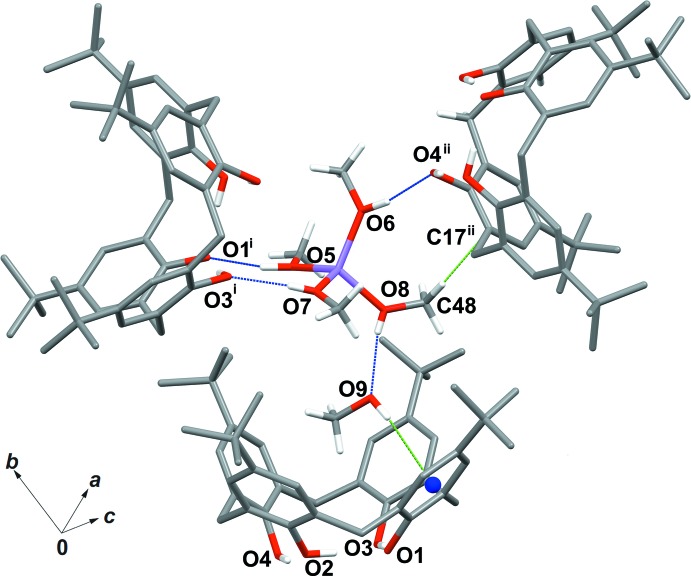
Hydrogen bonding (blue and green dotted lines) involving the [Li(CH_3_OH)_4_]^+^ complex and two adjacent calix[4]arene mol­ecules in the crystal structure. [Symmetry codes: (i) 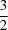 − *x*, 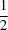 + *y*, 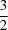 − *z*; (ii) 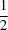 + *x*, 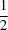 − *y*, 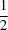 + *z*.]

Moreover, the coordinated methanol mol­ecules of [Li(CH_3_OH)_4_]^+^ further contribute to the stabilization of the complex in the structure, inter­acting with two other adjacent calixarene mol­ecules through hydrogen bonds and C—H⋯π inter­actions, as illustrated in Fig. 3 ▸ and Table 1 ▸. In particular, three of the coordinated methanol mol­ecules (C45–O5, C47–O7 and C46–O6), act as hydrogen-bond donors towards the hy­droxy groups at the lower rim of the macrocycle, namely O1^i^, O3^i^ and O4^ii^, respectively [symmetry codes: (i) −*x* + 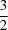, *y* + 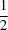, −*z* + 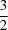; (ii) *x* + 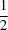, −*y* + 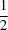, *z* + 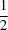]. In addition, the fourth coordinated methanol mol­ecule C48–O8 inter­acts with the aromatic-π electrons of a calixarene^ii^ *via* a C—H⋯π inter­action. The C48⋯C17^ii^ and C48—H64⋯C17^ii^ distances are 3.603 (4) and 2.628 Å, respectively, with a C48—H64⋯C17^ii^ angle of 173.3 (8)°.

**Figure 4:**
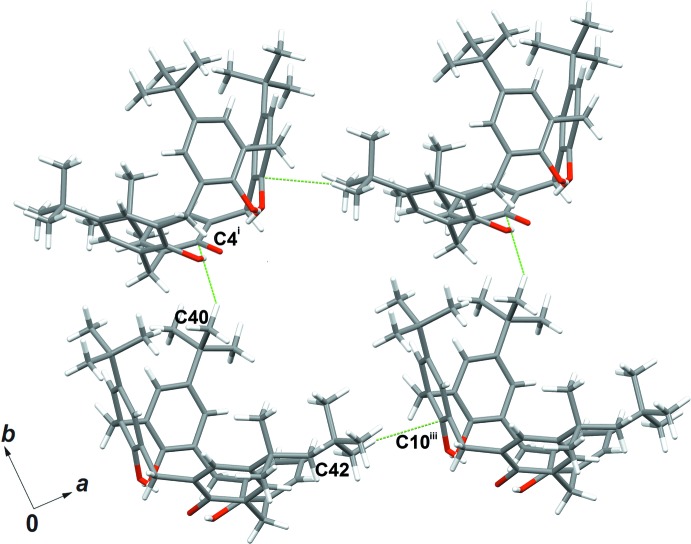
C—H⋯π inter­actions involving four adjacent calix[4]arene anions in the crystal structure. [Symmetry codes: (i) 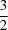 − *x*, 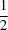 + *y*, 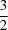 − *z*; (iii) 1 + *x*, *y*, *z*.]

Similarly, C—H⋯π inter­actions are also present between *tert*-butyl groups at the upper rim of the macrocycle and π-electrons of the aromatic walls of adjacent calix[4]arenes. In particular, Fig. 4 ▸ shows the spatial arrangement of four symmetry-related host mol­ecules [the C40⋯C4^i^ and C40—H41⋯C4^i^ distances are 3.498 (4) and 2.770 Å, respectively and the C40—H41⋯C4^i^ angle is 131.6 (5)° while the C42⋯C10^iii^ and C42—H46⋯C10^iii^ distances are 3.770 (5) and 2.828 Å, and the C42—H46⋯C10^iii^ angle is 161.7 (8)°; symmetry code: (iii) 1 + *x*, *y*, *z*].

## Database survey   

A search in the Cambridge Structural Database (Version 5.38, update May 2017; Groom *et al.*, 2016 ▸) based on a fragment comprising alkali metals and unsubstituted *p*-*tert*-butyl­calix[4]arenes, yielded the structures of several compounds.

In particular, inclusion complexes were found with: (i) lithium (ZESGIN, Bock *et al.*, 1995 ▸; RILNOP and RILNUV, Davidson *et al.*, 1997 ▸; YEMQIR, Dürr *et al.*, 2006 ▸; RUWVIO and RUWVOU, Gueneau *et al.*, 2003 ▸; NASWEJ, Hamada *et al.*, 1993 ▸; QUBJIH, Lee *et al.*, 2009 ▸; BASWEY, Hanna *et al.*, 2003 ▸); (ii) sodium (MODYIN, Guillemot *et al.*, 2002 ▸; NASSEF, Hamada *et al.*, 1993 ▸); (iii) potassium (MODYOT, Guillemot *et al.*, 2002 ▸; NASXUA, Hamada *et al.*, 1993 ▸; RUWVUA, Gueneau *et al.*, 2003 ▸; WUHVUQ and WUHWAX, Hanna *et al.*, 2002 ▸); (iv) rubidium (BASTUL, Hanna *et al.*, 2003 ▸); (v) cesium (JIVKEE, Harrowfield *et al.*, 1991 ▸).

In all the cases reported, the alkali metals inter­act with the calix[4]arene mol­ecules through the hy­droxy groups at the lower rim. The only exception is the complex with cesium, JIVKEE, in which the bare cation is placed well inside the cavity, on the quaternary axis passing through the macrocycle. The metal is involved in a polyhapto coordination with the four phenolate rings of the calix[4]arene, on which the negative charge is delocalized (Harrowfield *et al.*, 1991 ▸). This coordination mode is probably possible due to the dimensions of Cs^+^, which matches the cavity in size. In the case of lithium, the cationic radius is much smaller, hence a direct cavity–cation inter­action is less favoured, and the metal is either coordinating the hy­droxy oxygen atoms, or forming a second-sphere coordination supra­molecular complex, like in the title compound.

## Synthesis and crystallization   

To a white suspension of *p*-*tert*-butyl­calix[4]arene (2.00 g, 3.08 mmol) in THF (50 mL) was added LiH (0.245 g, 30.8 mmol), and a yellow suspension was obtained. The suspended mixture was stirred at room temperature for 5 h under a nitro­gen atmosphere, after which time, the mixture became a yellow clear solution. After quenching the excess of LiH with methanol, the solvent was removed *in vacuo*. The resulting yellow solid material was dissolved in methanol (80 mL) and the remaining insoluble matter was filtered off. The clear solution thus obtained was allowed to stand for several weeks to get colorless, thin plate-shaped crystals of the mol­ecular adduct of the title compound. IR (ATR): *ν* 2952.40 (*m*), 1478.65 (*s*), 1360.61 (*m*) cm^−1^; ^1^H NMR (300 MHz, CDCl_3_, TMS): δ 7.04 (*s*, 8H, Ar–H), 4.25 (*s*, 4H, –CH_2_–), 3.46 (*s*, 4H, –CH_2_–), 3.46 (*s*, 15H, –CH–, five methanol mol­ecules), 1.21 (*m*, 36H, *tert*-but­yl).

## Refinement details   

Crystal data, data collection and structure refinement details are summarized in Table 4 ▸. The C-bound H atoms were placed in calculated positions and refined using a riding model: C—H = 0.95–0.98 Å with *U*_iso_(H) = 1.5*U*_eq_(C-meth­yl) and 1.2*U*_eq_(C) for other H atoms. H atoms on O atoms were located in the difference-Fourier map and refined with *U*_iso_(H) = 1.5*U*_eq_(O).

## Supplementary Material

Crystal structure: contains datablock(s) I, global. DOI: 10.1107/S2056989018001834/xi2008sup1.cif

Structure factors: contains datablock(s) I. DOI: 10.1107/S2056989018001834/xi2008Isup2.hkl

CCDC reference: 1563055

Additional supporting information:  crystallographic information; 3D view; checkCIF report

## Figures and Tables

**Table 1:** Hydrogen-bond geometry (Å, °)

*D*—H⋯*A*	*D*—H	H⋯*A*	*D*⋯*A*	*D*—H⋯*A*
O8—H74⋯O9	0.67 (3)	2.01 (8)	2.673 (3)	167 (3)
O2—H68⋯O1	0.83 (3)	1.66 (4)	2.490 (2)	172 (3)
O3—H69⋯O1	0.89 (3)	1.64 (3)	2.520 (2)	169 (3)
O4—H70⋯O2	0.90 (3)	1.77 (3)	2.650 (2)	166 (3)
O5—H71⋯O1^i^	0.88 (4)	1.87 (4)	2.714 (3)	160 (4)
O6—H72⋯O4^ii^	0.94 (5)	1.81 (5)	2.732 (3)	165 (4)
O7—H73⋯O3^i^	0.79 (6)	1.91 (6)	2.676 (3)	163 (6)

Symmetry codes: (i) 
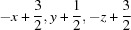
; (ii) 
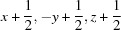
.

**Table 3:** Selected geometric parameters (Å, °)

Li1—O5	1.922 (6)	Li1—O7	1.903 (6)
Li1—O6	1.917 (6)	Li1—O8	1.922 (6)
			
O5—Li1—O6	107.2 (3)	O6—Li1—O7	112.3 (3)
O5—Li1—O7	111.3 (3)	O6—Li1—O8	109.9 (3)
O5—Li1—O8	111.0 (3)	O7—Li1—O8	105.3 (3)

**Table 2:** Conformation of the four aromatic walls of the calix[4]arene host (°) *A*–*D* are the mean planes passing through the four phenyl moieties of the host. The values reported are the angles formed with the mean plane passing through atoms O1–O4.

Plane	Angle
*A*	136.01 (6)
*B*	136.80 (6)
*C*	108.21 (6)
*D*	119.02 (6)

**Table 4:** Experimental details

Crystal data	
Chemical formula	[Li(CH_3_OH)_4_](C_44_H_55_O_4_)·CH_3_OH
*M*_r_	815.03
Crystal system, space group	Monoclinic, *P*2_1_/*n*
Temperature (K)	200
*a*, *b*, *c* (Å)	12.8434 (4), 20.0919 (6), 19.3168 (6)
β (°)	92.561 (2)
*V* (Å^3^)	4979.7 (3)
*Z*	4
Radiation type	Cu *K*α
μ (mm^−1^)	0.58
Crystal size (mm)	0.20 × 0.20 × 0.10

Data collection	
Diffractometer	Bruker APEXII CCD
Absorption correction	Multi-scan (*SADABS*; Bruker 2006 ▸)
*T*_min_, *T*_max_	0.893, 0.945
No. of measured, independent and observed [*I* > 2σ(*I*)] reflections	41849, 8251, 6715
*R*_int_	0.021
(sin θ/λ)_max_ (Å^−1^)	0.588

Refinement	
*R*[*F* ^2^ > 2σ(*F* ^2^)], *wR*(*F* ^2^), *S*	0.065, 0.203, 1.06
No. of reflections	8251
No. of parameters	557
H-atom treatment	H atoms treated by a mixture of independent and constrained refinement
Δρ_max_, Δρ_min_ (e Å^−3^)	1.46, −0.39

Computer programs: *APEX2* and *SAINT* (Bruker, 2006 ▸), *SHELXT* (Sheldrick, 2015*a* ▸), *SHELXL2014* (Sheldrick, 2015*b* ▸), *ORTEP-3 for Windows* (Farrugia, 2012 ▸), *Yadokari-XG* (Kabuto *et al.*, 2009 ▸) and *Mercury* (Macrae *et al.*, 2008 ▸).

## supplementary crystallographic information

### Crystal data

**Table:**

[Li(CH_3_OH)_4_](C_44_H_55_O_4_)·CH_3_OH	*F*(000) = 1776
*M**_r_* = 815.03	*D*_x_ = 1.087 Mg m^−^^3^
Monoclinic, *P*2_1_/*n*	Cu *K*α radiation, λ = 1.54178 Å
*a* = 12.8434 (4) Å	Cell parameters from 9823 reflections
*b* = 20.0919 (6) Å	θ = 3.2–63.8°
*c* = 19.3168 (6) Å	µ = 0.58 mm^−^^1^
β = 92.561 (2)°	*T* = 200 K
*V* = 4979.7 (3) Å^3^	Plane, colorless
*Z* = 4	0.20 × 0.20 × 0.10 mm

### Data collection

**Table:**

Bruker APEXII CCD diffractometer	8251 independent reflections
Radiation source: fine-focus sealed tube	6715 reflections with *I* > 2σ(*I*)
Detector resolution: 8.333 pixels mm^-1^	*R*_int_ = 0.021
φ and ω scans	θ_max_ = 65.0°, θ_min_ = 3.2°
Absorption correction: multi-scan (SADABS; Bruker 2006)	*h* = −14→14
*T*_min_ = 0.893, *T*_max_ = 0.945	*k* = −22→23
41849 measured reflections	*l* = −22→22

### Refinement

**Table:**

Refinement on *F*^2^	Primary atom site location: structure-invariant direct methods
Least-squares matrix: full	Secondary atom site location: difference Fourier map
*R*[*F*^2^ > 2σ(*F*^2^)] = 0.065	Hydrogen site location: mixed
*wR*(*F*^2^) = 0.203	H atoms treated by a mixture of independent and constrained refinement
*S* = 1.06	*w* = 1/[σ^2^(*F*_o_^2^) + (0.1063*P*)^2^ + 3.6084*P*] where *P* = (*F*_o_^2^ + 2*F*_c_^2^)/3
8251 reflections	(Δ/σ)_max_ < 0.001
557 parameters	Δρ_max_ = 1.46 e Å^−^^3^
0 restraints	Δρ_min_ = −0.39 e Å^−^^3^

### Special details

**Table:**

Geometry. All esds (except the esd in the dihedral angle between two l.s. planes) are estimated using the full covariance matrix. The cell esds are taken into account individually in the estimation of esds in distances, angles and torsion angles; correlations between esds in cell parameters are only used when they are defined by crystal symmetry. An approximate (isotropic) treatment of cell esds is used for estimating esds involving l.s. planes.
Refinement. Refinement was performed using all reflections. The weighted R-factor (wR) and goodness of fit (S) are based on F^2^. R-factor (gt) are based on F. The threshold expression of F^2^ > 2.0 sigma(F^2^) is used only for calculating R-factor (gt).

### Fractional atomic coordinates and isotropic or equivalent isotropic displacement parameters (Å^2^)

**Table:**

	*x*	*y*	*z*	*U*_iso_*/*U*_eq_	
C1	0.76784 (19)	0.08132 (12)	0.95506 (12)	0.0392 (5)	
C2	0.66560 (18)	0.09379 (12)	0.93080 (12)	0.0373 (5)	
H1	0.621091	0.118191	0.959486	0.045*	
C3	0.62623 (17)	0.07212 (11)	0.86666 (11)	0.0337 (5)	
C4	0.69117 (17)	0.03573 (11)	0.82392 (11)	0.0326 (5)	
C5	0.79496 (17)	0.02383 (11)	0.84604 (12)	0.0345 (5)	
C6	0.83058 (18)	0.04662 (12)	0.91089 (12)	0.0380 (5)	
H2	0.900898	0.038046	0.925538	0.046*	
C7	0.45550 (17)	0.26708 (12)	0.78857 (12)	0.0385 (5)	
C8	0.46863 (17)	0.26210 (13)	0.71766 (12)	0.0387 (5)	
H3	0.457513	0.300562	0.689690	0.046*	
C9	0.49727 (16)	0.20330 (12)	0.68618 (12)	0.0364 (5)	
C10	0.51265 (16)	0.14659 (12)	0.72660 (12)	0.0349 (5)	
C11	0.50132 (16)	0.14914 (12)	0.79826 (11)	0.0344 (5)	
C12	0.47390 (17)	0.20927 (12)	0.82756 (12)	0.0369 (5)	
H4	0.467306	0.211298	0.876300	0.044*	
C13	0.78349 (19)	0.26506 (12)	0.57083 (12)	0.0395 (5)	
C14	0.83391 (19)	0.20539 (12)	0.55992 (12)	0.0390 (6)	
H5	0.905410	0.206537	0.549319	0.047*	
C15	0.78502 (18)	0.14371 (12)	0.56372 (11)	0.0354 (5)	
C16	0.67899 (18)	0.14313 (12)	0.57703 (11)	0.0354 (5)	
C17	0.62574 (18)	0.20153 (12)	0.59032 (11)	0.0371 (5)	
C18	0.67867 (19)	0.26118 (13)	0.58751 (12)	0.0410 (6)	
H6	0.642613	0.301143	0.597261	0.049*	
C19	1.05823 (18)	0.09337 (12)	0.70485 (13)	0.0418 (6)	
C20	1.01017 (17)	0.05755 (12)	0.75598 (13)	0.0395 (5)	
H7	1.043514	0.054747	0.800787	0.047*	
C21	0.91492 (17)	0.02561 (11)	0.74393 (12)	0.0354 (5)	
C22	0.86546 (17)	0.03069 (11)	0.67883 (12)	0.0337 (5)	
C23	0.90887 (17)	0.06798 (12)	0.62654 (12)	0.0357 (5)	
C24	1.00575 (18)	0.09739 (12)	0.64050 (13)	0.0401 (6)	
H8	1.037292	0.121225	0.604494	0.048*	
C25	0.51496 (17)	0.08751 (12)	0.84342 (12)	0.0372 (5)	
H9	0.473547	0.093272	0.885011	0.045*	
H10	0.486068	0.048761	0.817385	0.045*	
C26	0.51156 (18)	0.20106 (13)	0.60839 (12)	0.0406 (6)	
H11	0.477998	0.160327	0.589089	0.049*	
H12	0.476077	0.239910	0.586407	0.049*	
C27	0.84858 (18)	0.08067 (12)	0.55860 (12)	0.0378 (5)	
H13	0.897651	0.085075	0.520772	0.045*	
H14	0.801769	0.042596	0.547653	0.045*	
C28	0.86781 (18)	−0.01488 (12)	0.80093 (12)	0.0375 (5)	
H15	0.925062	−0.033610	0.830930	0.045*	
H16	0.828803	−0.052668	0.779515	0.045*	
C29	0.8047 (2)	0.10237 (14)	1.02850 (13)	0.0491 (6)	
C30	0.7435 (4)	0.0613 (2)	1.08047 (17)	0.0999 (15)	
H17	0.668580	0.067370	1.070758	0.150*	
H18	0.761205	0.014078	1.075957	0.150*	
H19	0.761885	0.076280	1.127723	0.150*	
C31	0.7776 (3)	0.17521 (19)	1.0412 (2)	0.0817 (11)	
H20	0.702438	0.181772	1.033078	0.123*	
H21	0.797621	0.187096	1.089159	0.123*	
H22	0.815358	0.203520	1.009480	0.123*	
C32	0.9206 (3)	0.0945 (2)	1.04151 (19)	0.0893 (13)	
H23	0.940253	0.048049	1.033720	0.134*	
H24	0.957366	0.123369	1.009797	0.134*	
H25	0.939630	0.106944	1.089476	0.134*	
C33	0.4217 (2)	0.33149 (13)	0.82393 (14)	0.0483 (6)	
C34	0.3259 (3)	0.31832 (16)	0.86617 (17)	0.0646 (8)	
H26	0.268945	0.301671	0.835477	0.097*	
H27	0.343143	0.285070	0.901997	0.097*	
H28	0.304356	0.359772	0.888088	0.097*	
C35	0.3903 (3)	0.38581 (16)	0.77120 (18)	0.0744 (10)	
H29	0.333490	0.369450	0.740210	0.112*	
H30	0.367093	0.425437	0.795745	0.112*	
H31	0.450375	0.397248	0.743962	0.112*	
C36	0.5101 (3)	0.3574 (2)	0.8719 (2)	0.0909 (12)	
H32	0.571635	0.365840	0.844952	0.136*	
H33	0.488380	0.398774	0.893783	0.136*	
H34	0.527167	0.324072	0.907692	0.136*	
C37	0.8372 (2)	0.33242 (13)	0.56622 (14)	0.0502 (7)	
C38	0.7819 (4)	0.3747 (2)	0.5104 (3)	0.1056 (16)	
H35	0.816671	0.417941	0.507589	0.158*	
H36	0.784179	0.351932	0.465588	0.158*	
H37	0.709105	0.381352	0.522014	0.158*	
C39	0.9517 (3)	0.32673 (16)	0.54956 (18)	0.0671 (9)	
H38	0.982298	0.371323	0.547234	0.101*	
H39	0.988990	0.300901	0.585886	0.101*	
H40	0.957595	0.304301	0.504852	0.101*	
C40	0.8356 (3)	0.36674 (17)	0.6372 (2)	0.0764 (10)	
H41	0.869993	0.410165	0.634851	0.115*	
H42	0.763233	0.372949	0.650028	0.115*	
H43	0.872446	0.339017	0.672132	0.115*	
C41	1.1651 (2)	0.12642 (17)	0.71726 (16)	0.0569 (7)	
C42	1.2390 (3)	0.0983 (4)	0.6681 (3)	0.152 (3)	
H44	1.245849	0.050269	0.675822	0.228*	
H45	1.212602	0.106484	0.620456	0.228*	
H46	1.307207	0.119585	0.675508	0.228*	
C43	1.2088 (3)	0.1184 (3)	0.7914 (2)	0.0982 (14)	
H47	1.159602	0.137049	0.823511	0.147*	
H48	1.219424	0.070991	0.801543	0.147*	
H49	1.275553	0.141857	0.796839	0.147*	
C44	1.1537 (4)	0.2011 (2)	0.7058 (3)	0.1234 (19)	
H50	1.104797	0.219191	0.738495	0.185*	
H51	1.221716	0.222572	0.713330	0.185*	
H52	1.127111	0.209471	0.658278	0.185*	
C45	1.0638 (3)	0.4028 (2)	0.7522 (2)	0.0873 (12)	
H53	1.055124	0.421695	0.705518	0.131*	
H54	1.121242	0.425519	0.777618	0.131*	
H55	1.079539	0.355241	0.748939	0.131*	
C46	1.1121 (3)	0.48291 (18)	0.92075 (18)	0.0740 (9)	
H56	1.158468	0.496325	0.959936	0.111*	
H57	1.153669	0.471653	0.881086	0.111*	
H58	1.064767	0.519665	0.908156	0.111*	
C47	0.7645 (3)	0.3692 (2)	0.9612 (2)	0.0964 (13)	
H59	0.693686	0.387179	0.962822	0.145*	
H60	0.761040	0.320979	0.953826	0.145*	
H61	0.802949	0.378616	1.005018	0.145*	
C48	1.0529 (3)	0.2521 (2)	0.9146 (2)	0.0881 (12)	
H62	1.036956	0.204507	0.910682	0.132*	
H63	1.114548	0.262203	0.888369	0.132*	
H64	1.066664	0.263650	0.963430	0.132*	
C49	0.7898 (4)	0.2062 (3)	0.7671 (2)	0.1036 (15)	
H65	0.742500	0.244404	0.763110	0.18 (3)*	
H66	0.751149	0.165274	0.755859	0.28 (5)*	
H67	0.846004	0.211673	0.734910	0.18 (3)*	
Li1	0.9522 (4)	0.3821 (3)	0.8811 (3)	0.0634 (13)	
O1	0.65262 (12)	0.01180 (8)	0.76275 (8)	0.0357 (4)	
O2	0.53575 (13)	0.08851 (9)	0.69430 (8)	0.0407 (4)	
H68	0.572 (3)	0.0638 (16)	0.7202 (17)	0.061*	
O3	0.77306 (13)	−0.00130 (9)	0.66416 (9)	0.0417 (4)	
H69	0.736 (3)	0.0003 (15)	0.7021 (17)	0.063*	
O4	0.62688 (13)	0.08283 (9)	0.57403 (9)	0.0423 (4)	
H70	0.589 (3)	0.0795 (16)	0.6120 (18)	0.063*	
O5	0.97040 (17)	0.41161 (11)	0.78777 (11)	0.0619 (6)	
H71	0.933 (3)	0.440 (2)	0.762 (2)	0.093*	
O6	1.05402 (19)	0.42743 (12)	0.93941 (12)	0.0702 (6)	
H72	1.069 (3)	0.420 (2)	0.987 (3)	0.105*	
O7	0.8138 (2)	0.39801 (16)	0.90840 (17)	0.0986 (10)	
H73	0.783 (5)	0.430 (3)	0.894 (3)	0.148*	
O8	0.9716 (3)	0.28755 (14)	0.8890 (2)	0.1247 (15)	
H74	0.937 (6)	0.269 (4)	0.870 (4)	0.187*	
O9	0.8315 (5)	0.2023 (2)	0.8336 (3)	0.196 (3)	
H75	0.804567	0.170190	0.854065	0.295*	

### Atomic displacement parameters (Å^2^)

**Table:**

	*U*^11^	*U*^22^	*U*^33^	*U*^12^	*U*^13^	*U*^23^
C1	0.0394 (13)	0.0415 (13)	0.0365 (12)	0.0012 (10)	−0.0019 (10)	0.0006 (10)
C2	0.0352 (12)	0.0438 (13)	0.0331 (12)	0.0029 (10)	0.0024 (10)	0.0002 (10)
C3	0.0304 (11)	0.0381 (12)	0.0330 (11)	−0.0005 (9)	0.0043 (9)	0.0051 (9)
C4	0.0317 (12)	0.0350 (12)	0.0310 (11)	−0.0035 (9)	0.0007 (9)	0.0032 (9)
C5	0.0328 (12)	0.0364 (12)	0.0346 (12)	0.0008 (9)	0.0029 (9)	0.0034 (9)
C6	0.0309 (12)	0.0435 (13)	0.0392 (13)	−0.0001 (10)	−0.0021 (10)	0.0043 (10)
C7	0.0274 (11)	0.0481 (14)	0.0400 (13)	−0.0041 (10)	0.0021 (9)	0.0018 (10)
C8	0.0270 (11)	0.0491 (14)	0.0398 (13)	0.0001 (10)	0.0013 (9)	0.0074 (11)
C9	0.0224 (11)	0.0543 (14)	0.0325 (12)	0.0006 (10)	−0.0007 (9)	0.0037 (10)
C10	0.0209 (10)	0.0475 (14)	0.0363 (12)	−0.0020 (9)	−0.0003 (9)	−0.0004 (10)
C11	0.0198 (10)	0.0486 (14)	0.0348 (12)	−0.0016 (9)	0.0011 (9)	0.0038 (10)
C12	0.0266 (11)	0.0516 (14)	0.0326 (12)	−0.0015 (10)	0.0019 (9)	0.0025 (10)
C13	0.0439 (13)	0.0453 (14)	0.0296 (11)	0.0006 (11)	0.0045 (10)	−0.0008 (10)
C14	0.0356 (12)	0.0508 (15)	0.0310 (12)	−0.0005 (11)	0.0063 (9)	−0.0014 (10)
C15	0.0376 (12)	0.0441 (13)	0.0245 (10)	−0.0011 (10)	0.0031 (9)	−0.0027 (9)
C16	0.0358 (12)	0.0468 (14)	0.0235 (10)	−0.0020 (10)	−0.0003 (9)	−0.0011 (9)
C17	0.0351 (12)	0.0513 (14)	0.0249 (11)	0.0026 (10)	0.0000 (9)	0.0042 (10)
C18	0.0431 (14)	0.0479 (14)	0.0325 (12)	0.0077 (11)	0.0059 (10)	0.0022 (10)
C19	0.0285 (12)	0.0463 (14)	0.0510 (14)	−0.0005 (10)	0.0053 (10)	−0.0066 (11)
C20	0.0293 (12)	0.0465 (14)	0.0425 (13)	0.0035 (10)	0.0002 (10)	−0.0060 (11)
C21	0.0304 (12)	0.0363 (12)	0.0397 (12)	0.0032 (9)	0.0042 (9)	−0.0043 (10)
C22	0.0285 (11)	0.0342 (12)	0.0387 (12)	−0.0005 (9)	0.0038 (9)	−0.0062 (9)
C23	0.0313 (12)	0.0395 (13)	0.0370 (12)	0.0020 (10)	0.0074 (9)	−0.0058 (10)
C24	0.0321 (12)	0.0433 (13)	0.0458 (14)	−0.0014 (10)	0.0102 (10)	−0.0015 (10)
C25	0.0289 (12)	0.0491 (14)	0.0341 (12)	−0.0010 (10)	0.0059 (9)	0.0044 (10)
C26	0.0321 (12)	0.0570 (15)	0.0326 (12)	0.0068 (11)	−0.0011 (9)	0.0064 (11)
C27	0.0365 (12)	0.0457 (13)	0.0319 (12)	−0.0014 (10)	0.0083 (10)	−0.0067 (10)
C28	0.0332 (12)	0.0403 (13)	0.0390 (12)	0.0046 (10)	0.0020 (10)	0.0017 (10)
C29	0.0502 (15)	0.0572 (16)	0.0391 (14)	0.0016 (13)	−0.0063 (11)	−0.0075 (12)
C30	0.142 (4)	0.117 (3)	0.0401 (17)	−0.036 (3)	−0.008 (2)	−0.0003 (19)
C31	0.073 (2)	0.081 (2)	0.088 (2)	0.0166 (19)	−0.0279 (19)	−0.039 (2)
C32	0.066 (2)	0.129 (3)	0.069 (2)	0.030 (2)	−0.0309 (18)	−0.043 (2)
C33	0.0541 (16)	0.0450 (14)	0.0459 (14)	−0.0036 (12)	0.0046 (12)	−0.0010 (11)
C34	0.073 (2)	0.0594 (18)	0.0626 (18)	0.0134 (16)	0.0228 (16)	0.0010 (15)
C35	0.105 (3)	0.0514 (18)	0.069 (2)	0.0131 (18)	0.0228 (19)	0.0064 (15)
C36	0.085 (3)	0.084 (3)	0.102 (3)	−0.010 (2)	−0.015 (2)	−0.036 (2)
C37	0.0540 (16)	0.0452 (15)	0.0522 (15)	−0.0020 (12)	0.0103 (12)	0.0011 (12)
C38	0.103 (3)	0.078 (3)	0.134 (4)	−0.023 (2)	−0.020 (3)	0.058 (3)
C39	0.068 (2)	0.0579 (18)	0.078 (2)	−0.0197 (15)	0.0283 (17)	−0.0135 (16)
C40	0.083 (2)	0.0604 (19)	0.087 (2)	−0.0145 (17)	0.0283 (19)	−0.0277 (17)
C41	0.0343 (14)	0.073 (2)	0.0632 (18)	−0.0130 (13)	0.0023 (12)	−0.0065 (15)
C42	0.043 (2)	0.264 (7)	0.151 (5)	−0.049 (3)	0.038 (3)	−0.105 (5)
C43	0.054 (2)	0.143 (4)	0.096 (3)	−0.033 (2)	−0.0148 (19)	−0.004 (3)
C44	0.106 (4)	0.096 (3)	0.164 (5)	−0.055 (3)	−0.042 (3)	0.019 (3)
C45	0.064 (2)	0.102 (3)	0.096 (3)	0.032 (2)	0.003 (2)	0.001 (2)
C46	0.085 (2)	0.074 (2)	0.062 (2)	−0.0032 (19)	−0.0116 (17)	0.0150 (17)
C47	0.083 (3)	0.114 (3)	0.091 (3)	−0.004 (2)	−0.010 (2)	0.039 (3)
C48	0.104 (3)	0.086 (3)	0.074 (2)	0.034 (2)	0.000 (2)	0.003 (2)
C49	0.115 (4)	0.113 (4)	0.080 (3)	−0.023 (3)	−0.024 (3)	0.025 (2)
Li1	0.057 (3)	0.058 (3)	0.073 (3)	0.007 (2)	−0.015 (2)	0.014 (2)
O1	0.0329 (8)	0.0413 (9)	0.0329 (8)	−0.0036 (7)	0.0006 (6)	−0.0022 (6)
O2	0.0393 (9)	0.0495 (10)	0.0330 (9)	0.0039 (8)	−0.0017 (7)	0.0001 (7)
O3	0.0363 (9)	0.0506 (10)	0.0387 (9)	−0.0126 (7)	0.0052 (7)	−0.0054 (7)
O4	0.0384 (9)	0.0508 (10)	0.0376 (9)	−0.0085 (8)	0.0012 (7)	−0.0047 (7)
O5	0.0633 (13)	0.0662 (13)	0.0553 (12)	0.0252 (10)	−0.0069 (10)	0.0018 (10)
O6	0.0821 (16)	0.0774 (15)	0.0493 (12)	−0.0152 (12)	−0.0174 (11)	0.0159 (11)
O7	0.0702 (16)	0.105 (2)	0.122 (2)	0.0284 (15)	0.0196 (15)	0.0684 (19)
O8	0.109 (2)	0.0535 (15)	0.203 (4)	0.0076 (15)	−0.082 (2)	0.0104 (19)
O9	0.273 (6)	0.114 (3)	0.191 (4)	−0.084 (3)	−0.111 (4)	0.046 (3)

### Geometric parameters (Å, º)

**Table:**

C1—C6	1.387 (3)	C33—C34	1.529 (4)
C1—C2	1.397 (3)	C33—C35	1.535 (4)
C1—C29	1.535 (3)	C34—H26	0.9800
C2—C3	1.387 (3)	C34—H27	0.9800
C2—H1	0.9500	C34—H28	0.9800
C3—C4	1.405 (3)	C35—H29	0.9800
C3—C25	1.511 (3)	C35—H30	0.9800
C4—O1	1.349 (3)	C35—H31	0.9800
C4—C5	1.402 (3)	C36—H32	0.9800
C5—C6	1.392 (3)	C36—H33	0.9800
C5—C28	1.521 (3)	C36—H34	0.9800
C6—H2	0.9500	C37—C39	1.524 (4)
C7—C8	1.391 (3)	C37—C38	1.524 (5)
C7—C12	1.399 (3)	C37—C40	1.536 (4)
C7—C33	1.535 (4)	C38—H35	0.9800
C8—C9	1.386 (3)	C38—H36	0.9800
C8—H3	0.9500	C38—H37	0.9800
C9—C10	1.390 (3)	C39—H38	0.9800
C9—C26	1.523 (3)	C39—H39	0.9800
C10—O2	1.362 (3)	C39—H40	0.9800
C10—C11	1.399 (3)	C40—H41	0.9800
C11—C12	1.386 (3)	C40—H42	0.9800
C11—C25	1.520 (3)	C40—H43	0.9800
C12—H4	0.9500	C41—C42	1.484 (5)
C13—C14	1.383 (3)	C41—C44	1.522 (6)
C13—C18	1.400 (3)	C41—C43	1.523 (5)
C13—C37	1.524 (4)	C42—H44	0.9800
C14—C15	1.393 (3)	C42—H45	0.9800
C14—H5	0.9500	C42—H46	0.9800
C15—C16	1.397 (3)	C43—H47	0.9800
C15—C27	1.512 (3)	C43—H48	0.9800
C16—O4	1.384 (3)	C43—H49	0.9800
C16—C17	1.388 (3)	C44—H50	0.9800
C17—C18	1.380 (4)	C44—H51	0.9800
C17—C26	1.522 (3)	C44—H52	0.9800
C18—H6	0.9500	C45—O5	1.420 (4)
C19—C20	1.389 (4)	C45—H53	0.9800
C19—C24	1.390 (4)	C45—H54	0.9800
C19—C41	1.534 (4)	C45—H55	0.9800
C20—C21	1.392 (3)	C46—O6	1.397 (4)
C20—H7	0.9500	C46—H56	0.9800
C21—C22	1.387 (3)	C46—H57	0.9800
C21—C28	1.516 (3)	C46—H58	0.9800
C22—O3	1.368 (3)	C47—O7	1.354 (5)
C22—C23	1.394 (3)	C47—H59	0.9800
C23—C24	1.393 (3)	C47—H60	0.9800
C23—C27	1.515 (3)	C47—H61	0.9800
C24—H8	0.9500	C48—O8	1.340 (5)
C25—H9	0.9900	C48—H62	0.9800
C25—H10	0.9900	C48—H63	0.9800
C26—H11	0.9900	C48—H64	0.9800
C26—H12	0.9900	C49—O9	1.370 (6)
C27—H13	0.9900	C49—H65	0.9800
C27—H14	0.9900	C49—H66	0.9800
C28—H15	0.9900	C49—H67	0.9800
C28—H16	0.9900	Li1—O5	1.922 (6)
C29—C32	1.507 (4)	Li1—O6	1.917 (6)
C29—C31	1.527 (4)	Li1—O7	1.903 (6)
C29—C30	1.542 (5)	Li1—O8	1.922 (6)
C30—H17	0.9800	Li1—H74	2.29 (8)
C30—H18	0.9800	O2—H68	0.83 (3)
C30—H19	0.9800	O3—H69	0.89 (3)
C31—H20	0.9800	O4—H70	0.90 (3)
C31—H21	0.9800	O5—H71	0.88 (4)
C31—H22	0.9800	O6—H72	0.94 (5)
C32—H23	0.9800	O7—H73	0.79 (6)
C32—H24	0.9800	O8—H74	0.68 (8)
C32—H25	0.9800	O9—H75	0.8400
C33—C36	1.524 (5)		
			
C6—C1—C2	116.5 (2)	C34—C33—C7	110.0 (2)
C6—C1—C29	122.9 (2)	C35—C33—C7	112.0 (2)
C2—C1—C29	120.5 (2)	C33—C34—H26	109.5
C3—C2—C1	122.9 (2)	C33—C34—H27	109.5
C3—C2—H1	118.6	H26—C34—H27	109.5
C1—C2—H1	118.6	C33—C34—H28	109.5
C2—C3—C4	119.1 (2)	H26—C34—H28	109.5
C2—C3—C25	120.2 (2)	H27—C34—H28	109.5
C4—C3—C25	120.7 (2)	C33—C35—H29	109.5
O1—C4—C5	120.9 (2)	C33—C35—H30	109.5
O1—C4—C3	119.7 (2)	H29—C35—H30	109.5
C5—C4—C3	119.4 (2)	C33—C35—H31	109.5
C6—C5—C4	119.2 (2)	H29—C35—H31	109.5
C6—C5—C28	119.9 (2)	H30—C35—H31	109.5
C4—C5—C28	120.9 (2)	C33—C36—H32	109.5
C1—C6—C5	122.8 (2)	C33—C36—H33	109.5
C1—C6—H2	118.6	H32—C36—H33	109.5
C5—C6—H2	118.6	C33—C36—H34	109.5
C8—C7—C12	116.5 (2)	H32—C36—H34	109.5
C8—C7—C33	123.2 (2)	H33—C36—H34	109.5
C12—C7—C33	120.3 (2)	C13—C37—C39	112.9 (2)
C9—C8—C7	122.6 (2)	C13—C37—C38	109.8 (3)
C9—C8—H3	118.7	C39—C37—C38	108.5 (3)
C7—C8—H3	118.7	C13—C37—C40	108.8 (2)
C8—C9—C10	119.1 (2)	C39—C37—C40	105.9 (3)
C8—C9—C26	120.3 (2)	C38—C37—C40	110.9 (3)
C10—C9—C26	120.6 (2)	C37—C38—H35	109.5
O2—C10—C9	118.2 (2)	C37—C38—H36	109.5
O2—C10—C11	121.3 (2)	H35—C38—H36	109.5
C9—C10—C11	120.5 (2)	C37—C38—H37	109.5
C12—C11—C10	118.4 (2)	H35—C38—H37	109.5
C12—C11—C25	120.0 (2)	H36—C38—H37	109.5
C10—C11—C25	121.6 (2)	C37—C39—H38	109.5
C11—C12—C7	122.9 (2)	C37—C39—H39	109.5
C11—C12—H4	118.5	H38—C39—H39	109.5
C7—C12—H4	118.5	C37—C39—H40	109.5
C14—C13—C18	116.6 (2)	H38—C39—H40	109.5
C14—C13—C37	123.0 (2)	H39—C39—H40	109.5
C18—C13—C37	120.4 (2)	C37—C40—H41	109.5
C13—C14—C15	123.2 (2)	C37—C40—H42	109.5
C13—C14—H5	118.4	H41—C40—H42	109.5
C15—C14—H5	118.4	C37—C40—H43	109.5
C14—C15—C16	117.6 (2)	H41—C40—H43	109.5
C14—C15—C27	119.7 (2)	H42—C40—H43	109.5
C16—C15—C27	122.6 (2)	C42—C41—C44	110.0 (4)
O4—C16—C17	120.4 (2)	C42—C41—C43	110.0 (4)
O4—C16—C15	118.2 (2)	C44—C41—C43	105.7 (3)
C17—C16—C15	121.3 (2)	C42—C41—C19	109.2 (3)
C18—C17—C16	118.6 (2)	C44—C41—C19	108.9 (3)
C18—C17—C26	119.7 (2)	C43—C41—C19	113.0 (3)
C16—C17—C26	121.7 (2)	C41—C42—H44	109.5
C17—C18—C13	122.5 (2)	C41—C42—H45	109.5
C17—C18—H6	118.7	H44—C42—H45	109.5
C13—C18—H6	118.7	C41—C42—H46	109.5
C20—C19—C24	116.9 (2)	H44—C42—H46	109.5
C20—C19—C41	122.4 (2)	H45—C42—H46	109.5
C24—C19—C41	120.6 (2)	C41—C43—H47	109.5
C19—C20—C21	122.4 (2)	C41—C43—H48	109.5
C19—C20—H7	118.8	H47—C43—H48	109.5
C21—C20—H7	118.8	C41—C43—H49	109.5
C22—C21—C20	118.8 (2)	H47—C43—H49	109.5
C22—C21—C28	121.1 (2)	H48—C43—H49	109.5
C20—C21—C28	120.1 (2)	C41—C44—H50	109.5
O3—C22—C21	120.7 (2)	C41—C44—H51	109.5
O3—C22—C23	118.3 (2)	H50—C44—H51	109.5
C21—C22—C23	121.0 (2)	C41—C44—H52	109.5
C24—C23—C22	118.1 (2)	H50—C44—H52	109.5
C24—C23—C27	120.9 (2)	H51—C44—H52	109.5
C22—C23—C27	120.8 (2)	O5—C45—H53	109.5
C19—C24—C23	122.8 (2)	O5—C45—H54	109.5
C19—C24—H8	118.6	H53—C45—H54	109.5
C23—C24—H8	118.6	O5—C45—H55	109.5
C3—C25—C11	114.87 (18)	H53—C45—H55	109.5
C3—C25—H9	108.6	H54—C45—H55	109.5
C11—C25—H9	108.6	O6—C46—H56	109.5
C3—C25—H10	108.6	O6—C46—H57	109.5
C11—C25—H10	108.6	H56—C46—H57	109.5
H9—C25—H10	107.5	O6—C46—H58	109.5
C17—C26—C9	112.70 (18)	H56—C46—H58	109.5
C17—C26—H11	109.1	H57—C46—H58	109.5
C9—C26—H11	109.1	O7—C47—H59	109.5
C17—C26—H12	109.1	O7—C47—H60	109.5
C9—C26—H12	109.1	H59—C47—H60	109.5
H11—C26—H12	107.8	O7—C47—H61	109.5
C15—C27—C23	109.92 (18)	H59—C47—H61	109.5
C15—C27—H13	109.7	H60—C47—H61	109.5
C23—C27—H13	109.7	O8—C48—H62	109.5
C15—C27—H14	109.7	O8—C48—H63	109.5
C23—C27—H14	109.7	H62—C48—H63	109.5
H13—C27—H14	108.2	O8—C48—H64	109.5
C21—C28—C5	114.67 (19)	H62—C48—H64	109.5
C21—C28—H15	108.6	H63—C48—H64	109.5
C5—C28—H15	108.6	O9—C49—H65	109.5
C21—C28—H16	108.6	O9—C49—H66	109.5
C5—C28—H16	108.6	H65—C49—H66	109.5
H15—C28—H16	107.6	O9—C49—H67	109.5
C32—C29—C31	107.7 (3)	H65—C49—H67	109.5
C32—C29—C1	112.8 (2)	H66—C49—H67	109.5
C31—C29—C1	110.5 (2)	O5—Li1—O6	107.2 (3)
C32—C29—C30	111.3 (3)	O5—Li1—O7	111.3 (3)
C31—C29—C30	106.4 (3)	O5—Li1—O8	111.0 (3)
C1—C29—C30	108.0 (2)	O6—Li1—O7	112.3 (3)
C29—C30—H17	109.5	O6—Li1—O8	109.9 (3)
C29—C30—H18	109.5	O7—Li1—O8	105.3 (3)
H17—C30—H18	109.5	O7—Li1—H74	97 (2)
C29—C30—H19	109.5	O6—Li1—H74	126 (2)
H17—C30—H19	109.5	O8—Li1—H74	16 (2)
H18—C30—H19	109.5	O5—Li1—H74	103 (2)
C29—C31—H20	109.5	C10—O2—H68	111 (2)
C29—C31—H21	109.5	C22—O3—H69	108 (2)
H20—C31—H21	109.5	C16—O4—H70	108 (2)
C29—C31—H22	109.5	C45—O5—Li1	123.8 (3)
H20—C31—H22	109.5	C45—O5—H71	105 (3)
H21—C31—H22	109.5	Li1—O5—H71	130 (3)
C29—C32—H23	109.5	C46—O6—Li1	125.8 (2)
C29—C32—H24	109.5	C46—O6—H72	107 (3)
H23—C32—H24	109.5	Li1—O6—H72	127 (3)
C29—C32—H25	109.5	C47—O7—Li1	127.7 (3)
H23—C32—H25	109.5	C47—O7—H73	111 (4)
H24—C32—H25	109.5	Li1—O7—H73	120 (4)
C36—C33—C34	109.3 (3)	C48—O8—Li1	130.6 (3)
C36—C33—C35	109.1 (3)	C48—O8—H74	113 (7)
C34—C33—C35	106.5 (3)	Li1—O8—H74	115 (7)
C36—C33—C7	109.9 (2)	C49—O9—H75	109.5
			
C6—C1—C2—C3	1.2 (4)	C20—C21—C22—O3	178.3 (2)
C29—C1—C2—C3	−175.8 (2)	C28—C21—C22—O3	−0.6 (3)
C1—C2—C3—C4	0.3 (4)	C20—C21—C22—C23	−1.1 (3)
C1—C2—C3—C25	−179.8 (2)	C28—C21—C22—C23	179.9 (2)
C2—C3—C4—O1	177.1 (2)	O3—C22—C23—C24	−176.5 (2)
C25—C3—C4—O1	−2.8 (3)	C21—C22—C23—C24	3.0 (3)
C2—C3—C4—C5	−1.9 (3)	O3—C22—C23—C27	8.3 (3)
C25—C3—C4—C5	178.2 (2)	C21—C22—C23—C27	−172.2 (2)
O1—C4—C5—C6	−177.0 (2)	C20—C19—C24—C23	0.6 (4)
C3—C4—C5—C6	1.9 (3)	C41—C19—C24—C23	179.7 (2)
O1—C4—C5—C28	1.6 (3)	C22—C23—C24—C19	−2.8 (4)
C3—C4—C5—C28	−179.5 (2)	C27—C23—C24—C19	172.4 (2)
C2—C1—C6—C5	−1.1 (4)	C2—C3—C25—C11	95.4 (3)
C29—C1—C6—C5	175.7 (2)	C4—C3—C25—C11	−84.7 (3)
C4—C5—C6—C1	−0.4 (4)	C12—C11—C25—C3	−97.4 (2)
C28—C5—C6—C1	−179.0 (2)	C10—C11—C25—C3	84.6 (3)
C12—C7—C8—C9	0.7 (3)	C18—C17—C26—C9	−83.1 (3)
C33—C7—C8—C9	−178.8 (2)	C16—C17—C26—C9	96.4 (3)
C7—C8—C9—C10	0.7 (3)	C8—C9—C26—C17	102.9 (3)
C7—C8—C9—C26	−179.4 (2)	C10—C9—C26—C17	−77.1 (3)
C8—C9—C10—O2	176.83 (19)	C14—C15—C27—C23	76.7 (3)
C26—C9—C10—O2	−3.1 (3)	C16—C15—C27—C23	−98.7 (2)
C8—C9—C10—C11	−1.2 (3)	C24—C23—C27—C15	−85.5 (3)
C26—C9—C10—C11	178.8 (2)	C22—C23—C27—C15	89.5 (3)
O2—C10—C11—C12	−177.59 (19)	C22—C21—C28—C5	−89.8 (3)
C9—C10—C11—C12	0.4 (3)	C20—C21—C28—C5	91.3 (3)
O2—C10—C11—C25	0.4 (3)	C6—C5—C28—C21	−100.5 (3)
C9—C10—C11—C25	178.4 (2)	C4—C5—C28—C21	81.0 (3)
C10—C11—C12—C7	1.0 (3)	C6—C1—C29—C32	12.1 (4)
C25—C11—C12—C7	−177.0 (2)	C2—C1—C29—C32	−171.1 (3)
C8—C7—C12—C11	−1.6 (3)	C6—C1—C29—C31	132.7 (3)
C33—C7—C12—C11	177.9 (2)	C2—C1—C29—C31	−50.6 (4)
C18—C13—C14—C15	1.1 (3)	C6—C1—C29—C30	−111.3 (3)
C37—C13—C14—C15	−179.7 (2)	C2—C1—C29—C30	65.4 (4)
C13—C14—C15—C16	2.1 (3)	C8—C7—C33—C36	−113.7 (3)
C13—C14—C15—C27	−173.5 (2)	C12—C7—C33—C36	66.8 (3)
C14—C15—C16—O4	173.58 (19)	C8—C7—C33—C34	125.9 (3)
C27—C15—C16—O4	−10.9 (3)	C12—C7—C33—C34	−53.5 (3)
C14—C15—C16—C17	−4.0 (3)	C8—C7—C33—C35	7.8 (4)
C27—C15—C16—C17	171.6 (2)	C12—C7—C33—C35	−171.7 (3)
O4—C16—C17—C18	−175.0 (2)	C14—C13—C37—C39	−0.9 (4)
C15—C16—C17—C18	2.5 (3)	C18—C13—C37—C39	178.4 (2)
O4—C16—C17—C26	5.6 (3)	C14—C13—C37—C38	120.3 (3)
C15—C16—C17—C26	−176.9 (2)	C18—C13—C37—C38	−60.5 (4)
C16—C17—C18—C13	0.9 (3)	C14—C13—C37—C40	−118.2 (3)
C26—C17—C18—C13	−179.6 (2)	C18—C13—C37—C40	61.1 (3)
C14—C13—C18—C17	−2.7 (3)	C20—C19—C41—C42	120.0 (4)
C37—C13—C18—C17	178.1 (2)	C24—C19—C41—C42	−58.9 (5)
C24—C19—C20—C21	1.3 (4)	C20—C19—C41—C44	−119.8 (4)
C41—C19—C20—C21	−177.7 (2)	C24—C19—C41—C44	61.2 (4)
C19—C20—C21—C22	−1.1 (3)	C20—C19—C41—C43	−2.7 (4)
C19—C20—C21—C28	177.8 (2)	C24—C19—C41—C43	178.3 (3)

### Hydrogen-bond geometry (Å, º)

**Table:**

*D*—H···*A*	*D*—H	H···*A*	*D*···*A*	*D*—H···*A*
O8—H74···O9	0.67 (3)	2.01 (8)	2.673 (3)	167 (3)
O2—H68···O1	0.83 (3)	1.66 (4)	2.490 (2)	172 (3)
O3—H69···O1	0.89 (3)	1.64 (3)	2.520 (2)	169 (3)
O4—H70···O2	0.90 (3)	1.77 (3)	2.650 (2)	166 (3)
O5—H71···O1^i^	0.88 (4)	1.87 (4)	2.714 (3)	160 (4)
O6—H72···O4^ii^	0.94 (5)	1.81 (5)	2.732 (3)	165 (4)
O7—H73···O3^i^	0.79 (6)	1.91 (6)	2.676 (3)	163 (6)

Symmetry codes: (i) −*x*+3/2, *y*+1/2, −*z*+3/2; (ii) *x*+1/2, −*y*+1/2, *z*+1/2.
